# Dysregulation of POPDC1 promotes breast cancer cell migration and proliferation

**DOI:** 10.1042/BSR20171039

**Published:** 2017-11-21

**Authors:** Johanna Ndamwena Amunjela, Steven John Tucker

**Affiliations:** School of Medicine, Medical Sciences and Nutrition, University of Aberdeen, Aberdeen AB25 2ZD, Scotland, U.K.

**Keywords:** breast cancer, cAMP, cell migration, cell proliferation, POPDC1

## Abstract

Breast cancer subtypes such as triple-negative that lack the expression of oestrogen receptor (ER), progesterone receptor (PR) and human epidermal growth factor 2 receptor (HER2), remain poorly clinically managed due to a lack of therapeutic targets. This necessitates identification and validation of novel targets. Suppression of Popeye domain-containing protein 1 (POPDC1) is known to promote tumorigenesis and correlate to poor clinical outcomes in various cancers, and also promotes cardiac and skeletal muscle pathologies. It remains to be established whether POPDC1 is dysregulated in breast cancer, and whether overcoming the dysregulation of POPDC1 could present a potential therapeutic strategy to inhibit breast tumorigenesis. We assessed the potential of POPDC1 as a novel target for inhibiting breast cancer cell migration and proliferation. POPDC1 was significantly suppressed with reduced cell membrane localization in breast cancer cells. Furthermore, functional suppression of POPDC1 promoted breast cancer cell migration and proliferation, which were inhibited by POPDC1 overexpression. Finally, cAMP interacts with POPDC1 and up-regulates its expression in breast cancer cells. These findings suggest that POPDC1 plays a role in breast tumorigenesis and represents a potential therapeutic target or biomarker in breast cancer medicine.

## Introduction

Breast cancer is the second leading cause of cancer-related morbidity and mortality globally [1]. Despite major progress in the development of new breast cancer treatments and in the clinical management of the disease over the years, these have been met with major challenges including drug resistance [2–5]. Furthermore, breast cancer is a molecularly heterogeneous disease with subtypes that have proven challenging to target and which display varying levels of susceptibility to therapeutic agents [4,6,7]. This necessitates identification and validation of novel therapeutic targets that can be targeted to deliver maximum clinical efficacy with minimum side effects especially in poorly managed breast cancer subtypes. Breast cancer can be classified on a molecular basis into five subtypes: luminal A, luminal B, HER2 positive, claudin-low and basal-like, according to the expression levels of oestrogen receptors (ER), progesterone receptors (PR) and human epidermal growth factor receptor 2 (HER2) in the cell. Luminal A tumours are ER^+^, PR^+/−^ and HER2^−^, luminal B tumours are ER^+^, PR^+/−^ and HER2^+^, HER2 positive tumours are ER^−^, PR^−^ and HER2^+^. Basal-like tumours and claudin-low tumours are subclasses of triple negative breast cancer and are ER^−^, PR^−^ and HER2^−^ [8]. Basal-like tumours are aggressive, have poor prognosis and are more challenging to therapeutically target due to a lack of hormone receptor expression. In contrast, luminal A tumours have a more positive prognosis, are less aggressive and easier to treat due to the expression of hormone receptors [6,8]. Here we assess the potential of Popeye domain-containing protein 1 (POPDC1) (also known as blood vessel epicardial substance (BVES)), as a novel target for inhibiting cell migration and proliferation in breast cancer. We use a non-malignant breast cell line (MCF10A), a less aggressive luminal A breast cancer cell line (MCF7) and aggressive breast cancer cell lines MDA231 (also known as MDA-MB-231) which is triple-negative and the HER2+ cell line (SKBR3).

POPDC1 (also known as BVES), is a transmembrane protein encoded by the *POPDC1* gene and is thought to be a tumour suppressor that is dysregulated to promote malignant cell behaviour. Loss of POPDC1 expression has been correlated with enhanced cancer cell proliferation, migration, invasion, metastasis, drug resistance and poor patient survival in various human cancers [9–13]. Suppression of POPDC1 has further been shown to promote cell migration and invasion in hepatocellular carcinoma, and to promote tumorigenesis in colorectal cancer [9,11]. In addition, loss of POPDC1 has been shown to promote colorectal cancer tumorigenesis via activation of c-Myc regulated networks and activation of Wnt signalling [12]. Although the exact functional mechanisms of POPDC1 are poorly understood, the known roles and correlations between POPDC1 with cancer and cardiovascular diseases have been recently reviewed [14,15].

POPDC1 belongs to the *POPDC* gene family which has three isoforms: *POPDC1, POPDC2* and *POPDC3* which encode the POPDC1, POPDC2 and POPDC3 proteins respectively. POPDC proteins are transmembrane proteins normally tethered to the cell membrane as a dimer held together by a disulphide bond [13,16,17]. They contain an extracellular amino terminus, three transmembrane domains and a cytoplasmic Popeye domain which binds cyclic nucleotides. The Popeye domain is evolutionary conserved and has been shown to bind cAMP with high affinity. The binding of cyclic adenosine monophosphate (cAMP) to the Popeye domain is thought to induce structural changes in POPDC1 that affects protein function [16,18]. The signalling cascade downstream of POPDC1 has not yet been determined. Although the role of POPDC1 in breast cancer tumorigenesis remains to be established, POPDC1 presents a realistically druggable target for various reasons. Firstly, POPDC1 possesses a novel Popeye domain (PFAM: PF04831), which has not been identified in any other protein outside the POPDC protein family [14,18–20]. The Popeye domain has been linked to POPDC protein functions such as binding cAMP and maintenance of epithelial integrity [15,21]. For example, truncation of the protein following introduction of an early stop codon has been shown to prevent localization of POPDC1 to the cell membrane and prevent POPDC1-mediated tight junction maintenance [21]. Hence the Popeye domain can be targeted to potentially induce effects specific to POPDC signalling with less ubiquitous side effects than targeting molecules such as cAMP. Secondly, the reduced expression of POPDC1 consistently correlates to tumorigenesis in various cancers and to the promotion of cardiovascular and muscular pathologies [14–16,19]. POPDC1 can therefore potentially be targeted to stabilize the protein, prevent loss of function and withdrawal from the membrane to reduce pathological consequences.

Cyclic adenosine monophosphate (cAMP) is a second messenger molecule involved in signal transduction of, for example, G-protein-coupled receptors. cAMP is synthesized when the enzyme adenylyl cyclase catalyses the conversion of adenosine triphosphate (ATP) to cAMP. In breast cancer, elevation of intracellular cAMP concentrations has been shown to promote apoptosis and inhibit cell migration and invasion [22,23]. In addition, the elevation of intracellular cAMP concentrations has been shown to inhibit breast tumour growth in mouse xenografts [24]. However, it remains to be established whether cAMP regulates POPDC1 in breast cancer, and whether POPDC1 is involved in cAMP-mediated inhibition of cell migration, invasion and tumour growth.

We hypothesize that dysregulation of POPDC1 promotes malignant phenotypes in breast cancer and that restoration of POPDC1 can potentially inhibit cell migration and proliferation, and revert cells to a less malignant phenotype. To test this hypothesis, we firstly determined the expression levels of POPDC1 in breast cancer cells in comparison with normal breast cells. Secondly, we assessed the effects of loss and gain of POPDC1 functions on breast cancer cell migration and proliferation. Thirdly, we determined whether cAMP interacts with, and regulates the levels of POPDC1 in breast cancer cells. Finally, we assessed whether cAMP-mediated inhibition of cell migration and proliferation is potentially facilitated via POPDC1 signalling.

This paper demonstrates firstly the suppression and loss of cell membrane localization of POPDC1 in breast cancer cells. Secondly, suppression of POPDC1 promotes cell migration and proliferation in breast cancer cells, which are significantly inhibited by the overexpression of POPDC1. Thirdly, cAMP interacts with and regulates POPDC1 expression in breast cancer cells and finally, cAMP-mediated inhibition of breast cancer cell migration and proliferation is potentially mediated via POPDC1 signalling.

## Materials and methods

### Cell culture

MCF10A cells (ATCC) were grown in mammary epithelial cell growth medium bullet kit (Lonza). MCF7 cells (ATCC), MDA231 cells (ATCC) and SKBR3 cells (ATCC) were grown in Dulbecco’s Modified Eagle’s medium (DMEM) (Sigma) supplemented with 10% foetal calf serum (FCS), 2 mM L-glutamine (PAA Laboratories) and 1% penicillin/streptomycin (penicillin 10,000 units/ml; streptomycin 10 mg/ml) (BioSera). Cells were grown at 37°C, 5% CO_2_ in a sterile humidified incubator and passaged at ≥80% confluence. MCF10A is a non-malignant breast cell line and MCF7 is a luminal A breast cancer cell line classified as ER+, PR+/- and HER2-. MDA231 is a basal-like triple negative breast cancer cell line classified as ER-, PR- and HER2- and SKBR3 is a HER2+ cell line classified as ER-, PR- and HER2+.

### Western blot

Cells were lysed in RIPA (radioimmunoprecipitation assay) buffer containing 50 mM Tris/HCl at pH 8, 150 mM sodium chloride, 0.1% Triton-X 100, 0.1% sodium dodecyl sulphate, 0.5% sodium deoxycholate, 0.5% Nonidet P-40, 1% protease inhibitor (Sigma) and 0.1% phosphatase inhibitor (Sigma). Lysates were subsequently homogenized through a 25 gauge needle. Gel electrophoresis was performed on a 10% polyacrylamide gel and proteins were transferred onto a nitrocellulose membrane (Biorad). Antibodies used for immunoblotting included rabbit anti-Popdc1 diluted 1:100 (Abcam), rabbit anti-phospho-CREB (Ser133) (Cell Signalling Technology) diluted 1:500, goat anti-rabbit conjugated with horseradish peroxidase diluted 1:500 (Abcam) and mouse anti-β-actin-peroxidase diluted 1:10000 (Sigma). Pull-down assay membranes were stained with anti-Popdc1 diluted 1:30 (Abcam), anti-β-actin-peroxidase diluted 1:5000 (Sigma) and goat anti-rabbit IgG conjugated with horseradish peroxidase diluted 1:100 (Abcam). Protein bands were detected with an ECL chemiluminescence reaction kit (ThermoScientific).

### Immunocytochemistry

Sterile glass coverslips were pre-coated with 10 μg/ml poly-l-lysine (PLL) and dried under sterile conditions. A total of 2 × 10^4^ cells were seeded on sterile PLL-coated glass coverslips and incubated at 37°C, 5% CO_2_ in a humidified incubator overnight. Cells were fixed onto coverslips with 4% paraformaldehyde for 20 min prior to 10-min permeabilization with 0.5% Triton X-100. Coverslips were rinsed three times in PBS prior to blocking, and after each step in the staining procedure. Non-specific binding sites were blocked with 5% BSA dissolved in PBS, for 30 min at room temperature. Cells were subsequently stained overnight at 4°C with CF-555 Phalloidin (Biotium) diluted 1:40 in PBS. Primary antibody staining with POPDC1 antibody (Sigma) (diluted 1:100) was subsequently performed with overnight incubation at 4°C. Secondary antibody staining was performed with Biotin-SP-conjugated goat anti-rabbit secondary antibody diluted 1:250 (Abcam) for 1 h at room temperature. Tertiary antibody staining was subsequently performed with Streptavidin-fluorescein diluted 1:150 (Abcam) for 30 min at room temperature. Counterstaining with 0.1 μg/ml DAPI dilactate nuclear probe (Sigma) was performed in the dark at room temperature for 10 min. Coverslips were mounted onto microscope slides with 1,4-diazabicyclo[2.2.2]octane (DABCO) mountant (Sigma), and stored in the dark. Confocal images were captured in single confocal plane with a Zeiss LSM700 confocal microscope at 63× magnification and processed using Zeiss Zen Black software. POPDC1 fluorescence intensity was measured with the ImageJ software. To quantitatively compare cell membrane expression of POPDC1 relative to overall expression in individual cells, fluorescence from the POPDC1 protein was measured per μm^2^ in the membrane and also across the whole cell. This was then presented as a comparative ratio of membrane fluorescence intensity/overall cell fluorescence intensity.

### Protein suppression with siRNA

A total of 2 × 10^4^ cells per/ml were grown in complete medium 24 h prior to transient transfection with POPDC1 siRNA (Qiagen) or scrambled siRNA (Qiagen). Cells were incubated in serum-free medium 30 min prior to transfection. siRNA was pre-mixed with Lipofectamine 2000^®^ (Invitrogen) in serum-free medium for 20 min and added to the cells. Complete medium was added to the cells 3 h post-transfection to minimize toxicity to the cells. Boyden chamber assay, Alamar Blue assay and Western blot analyses were performed 36 h post-transfection. Cells were starved overnight prior to performing the Alamar Blue assay.

### Generation of stable cell lines

*mPOPDC1* cDNA containing a G418 resistance cassette was kindly gifted by Professor Thomas Brand (Imperial College London). Plasmid DNA was transfected into MCF7, MDA231 and SKBR3 cells using Amaxa Nucleofector™ II (Lonza) to generate cell lines stably overexpressing POPDC1; MCF7 POP1++, MDA POP1++ and SKBR3 POP1++. Selection of stably transfected cells was performed with 1 mg/ml Geneticin sulphate (Biovision) (concentration verified using a kill curve) for 21 days. G418 (0.5 mg/ml) was added to complete growth medium to maintain selection of MCF7 POP1++, MDA231 POP1++ SKBR3 POP1 ++ cell lines thereafter.

### Boyden migration assay

Boyden chamber migration assays were performed to assess cell migration using 8 μm pore polycarbonate membranes (Neuro Probe Inc.). For experiments where the effects of POPDC1 knockdown and POPDC1 overexpression on cell migration were assessed, lower wells of the Boyden chamber were loaded with serum-free medium lacking any migrant solution to ensure that the observed difference in migration was due to the effects of the difference in POPDC1 expression levels. For experiments where the effects of cAMP on cell migration were measured, lower wells were loaded with 60 µM Sp-8-Br-cAMPS (in serum-free medium) for the test samples and simply serum-free medium for the control samples. Cells were suspended in serum-free medium to a density of 2 × 10^5^ cells/ml prior to loading into upper wells of the chamber. After a 3-h incubation at 37°C and 5% CO_2_ in a sterile humidified incubator, non-migrated cells were scraped from the membrane surface and migrated cells were stained with a Diff Quik staining kit (Medion Diagnostix AG). Stained cells were subsequently counted using a light microscope and representative images were captured using Volocity software at 100× magnification.

### AlamarBlue^®^ cell proliferation assay

A total of 2 × 10^3^ cells/well were seeded in 96-well plates and were serum starved overnight prior to drug treatment. AlamarBlue^®^ dye (Thermoscientific) was added to each well at a final concentration of 10% (v/v) and cells were incubated for 4 h at 37°C and 5% CO_2_ in a sterile humidified incubator. Fluorimetric quantitation of the reduced AlamarBlue^®^ dye was measured with a Biotek Synergy™ HT multi-detection microplate reader and Gen5 software at 530 nm excitation and 590 nm emission wavelengths.

### Pull-down assay

Cells were lysed in NP-40 lysis buffer containing 50 mM Tris at pH 7.5, 150 mM sodium chloride, 1% Nonidet P-40, 5% glycerol, 1% protease inhibitor and 0.1% phosphatase inhibitor. Lysates were subsequently homogenized through a 25 gauge needle. Adenosine 3′,5′-cyclic monophosphate (cAMP) agarose beads (Sigma) were mixed with phosphate buffered saline (PBS) at pH 7.4 to form a 50% slurry. cAMP agarose bead slurry (400 μl) was centrifuged in a Pierce^®^ spin cup (Thermo Scientific) at 1000×***g*** for 1 min. Lysate (150 μl) was diluted with 150 μl of PBS at pH 7.4 and gently mixed with cAMP agarose beads in a spin cup and incubated for 6 h at 4°C with gentle agitation. The mixture was subsequently centrifuged at 1000×***g*** for 1 min to filter out the liquid phase of the lysate. The beads were washed with 150 μl of PBS at pH 7.4 and subjected to centrifugation at 1000×***g*** for 1 min to wash off unbound elements from the beads. Four additional washes were subsequently performed. To elute specifically bound proteins, beads were gently mixed with 150 µl of elution buffer (0.1 M glycine at pH 2.5) and centrifuged at 1000×***g*** for 1 min. Four elution steps were performed separately. Neutralization buffer (5 μl; 1 M Tris at pH 8.0) was added to each eluted fraction for pH neutralization. Western blot analysis was performed on the lysate, wash and elution fractions as previously described.

### cAMP treatment

Cells were grown to 70% confluence in 6 or 9 cm tissue culture dishes prior to Sp-8-Br-cAMPS (Biolog) treatment. Optimal drug concentration selection was performed with a range of Sp-8-Br-cAMPS concentrations; 20, 40 and 60 µM diluted in serum-free DMEM. Cells were incubated at 37°C and 5% CO_2_ in a sterile humidified incubator for 1 h. Time course analysis of the effects of Sp-8-Br-cAMPS on POPDC1 expression was performed with 60 µM Sp-8-Br-cAMPS diluted in serum-free DMEM, over 1, 3 and 6 h incubation periods at 37°C and 5% CO_2_ in a sterile humidified incubator. Control samples were treated with serum-free medium. To assess whether up-regulation of POPDC1 by cAMP pre-treatment affected cell proliferation, cells were treated with 60 µM Sp-8-Br-cAMPS for 1 h, prior to replacement of the treatment solution with a fresh 60 µM Sp-8-Br-cAMPS solution and incubation for 24 h. This two-phase treatment approach initially up-regulates POPDC1 (1st Sp-8-Br-cAMPS application), and then assesses the functional impact of the high intracellular cAMP levels provided by the second Sp-8-Br-cAMPS application on the up-regulated POPDC1 levels. Cell lysis and Western blot analysis were performed as described above.

### Statistical analysis

Unpaired two-tailed Student’s *t*-test was performed on datasets with two comparison groups and one-way ANOVA with Dunnett’s *post hoc* tests were performed on comparisons of more than two groups of data. A *P*-value ≤ 0.05 was considered statistically significant. Statistical significance was classified as follows: *P≤0.05, **P≤0.01 and ***P≤0.001.

## Results

### The expression of POPDC1 is suppressed in breast cancer cell lines in comparison with non-malignant cells

**Figure 1:**
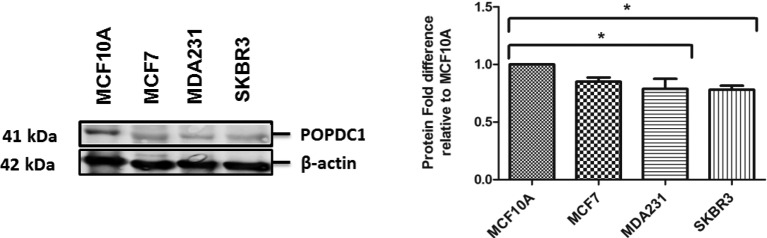
POPDC1 is suppressed in breast cancer cells Western blot analysis of POPDC1 expression in breast cell lines. POPDC1 is expressed at lower levels in malignant MDA231 and SKBR3 cells in comparison with non-malignant MCF10A breast cells (*n*=3). Graph represents densitometric quantification of protein bands. Protein bands were normalized by calculating the POPDC1/β-actin ratio. Normalized ratios for each cell line were expressed as a ratio relative to MCF10A to show protein fold differences for each cell line relative to the MCF10A cells. Comparisons of protein fold ratios were conducted using ANOVA with Dunnett’s *post hoc* test; *P≤0.05.

Suppression of POPDC1 at protein and mRNA level has been observed in various cancers [11–13,25]. We assessed if POPDC1 was suppressed in breast cancer cells in comparison with non-malignant breast cells. Interestingly, POPDC1 was significantly suppressed in the more aggressive breast cancer subtypes, MDA231 triple-negative cells and SKBR3 HER2+ cells, in comparison with non-malignant MCF10A cells (Figure 1).

### Cell membrane localization of POPDC1 is reduced in breast cancer cell lines

**Figure 2:**
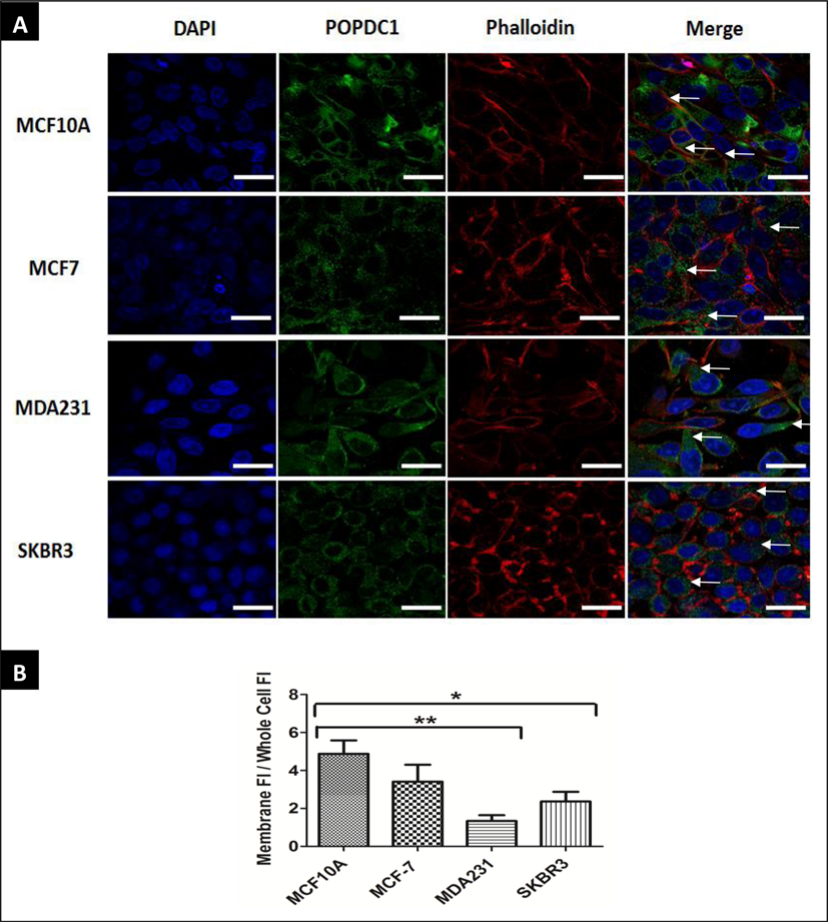
POPDC1 membrane localization is reduced in cancer cells (**A**) Cell membrane expression of POPDC1 is reduced in breast cancer cell lines. Immunocytochemical analysis of cell membrane expression of POPDC1 in breast cancer cells. Cells were counterstained with CF-555 Phalloidin (red) for visualization of filamentous actin on the cytoskeleton and nuclear probe DAPI (blue). In MCF10A non-malignant cells, regions of high POPDC1 (green) localization on the plasma membrane (white arrows) are observed. Plasma membrane expression of POPDC1 is reduced in MDA231 and SKBR3 cells (white arrows indicate POPDC1 located in other cellular locations in these images); scale bars = 20 µm. (**B**) Quantitative analysis of the membrane expression of POPDC1 in different cell lines. Membrane POPDC1 fluorescence intensity per μm^2^ was measured for individual cells along with fluorescence intensity per μm^2^ across the whole cell. This was then converted to a ratio of fluorescence intensity in the membrane/fluorescence intensity across the entire cell. Membrane expression of POPDC1 is significantly reduced in MDA231 and SKBR3 cells compared with non-malignant MCF10A cells (*n*=4). Comparisons were conducted using ANOVA with Dunnett’s; *P≤0.05, **P≤0.01.Data are presented as mean ratio ± SEM.

Cell membrane localization of POPDC1 is known to be observed in various epithelial cells [26], so we next assessed if POPDC1 localization is dysregulated in malignant MCF7 and MDA231 breast cells in comparison with non-malignant MCF10A breast cells (Figure 2). POPDC1 (green) co-localized with Phalloidin (red) and is localized to the cell membrane of MCF10A non-malignant cells suggesting that POPDC1 is expressed primarily on the cell membrane of MCF10A cells (Figure 2). This is consistent with its junctional function, and role in maintaining epithelial barrier integrity. Cell membrane expression of POPDC1 is significantly reduced in MDA231 cells and SKBR3 cells suggesting that the membrane localization of POPDC1 is reduced in triple negative and HER2+ breast cancer cells. Loss of POPDC1 expression on the cell membrane could contribute to enhanced migration and malignant development by loss of the POPDC1 role in the maintenance of epithelial integrity, loss of tight junction maintenance and general molecular instability.

### Suppression of POPDC1 promotes cell migration and proliferation in breast cancer cells

**Figure 3:**
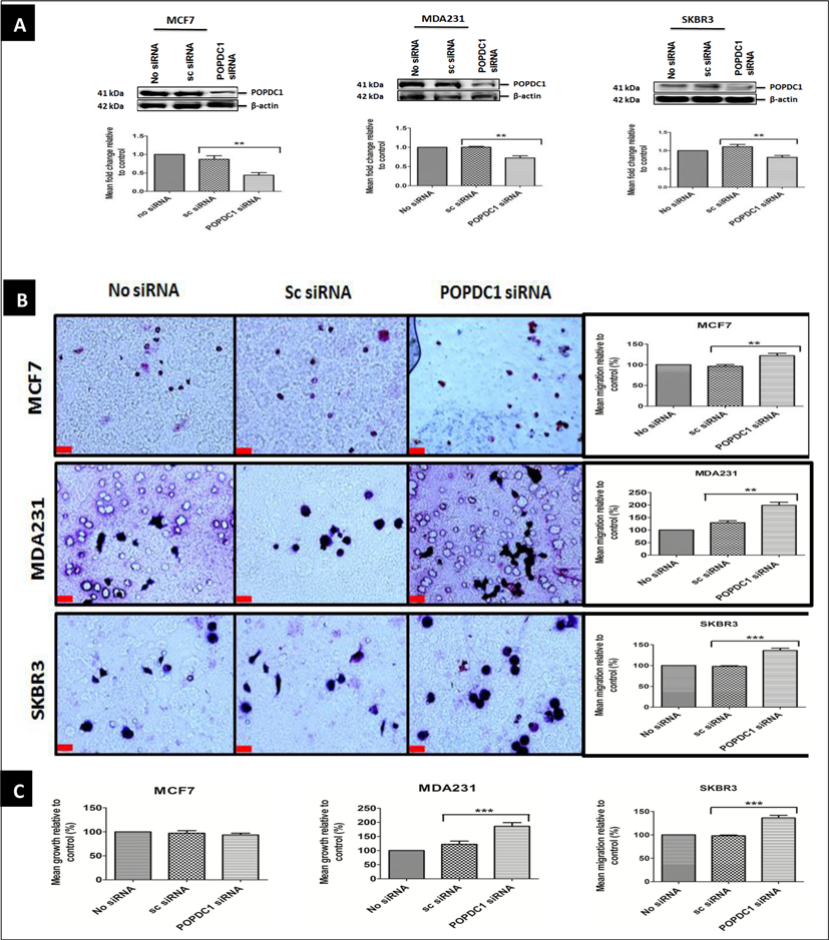
Suppression of POPDC1 promotes cell migration and proliferation in breast cancer cells (**A**) Western blot analysis of POPDC1 expression following transfection with POPDC1 siRNA confirmed POPDC1 suppression in MCF7 (*n*=3), MDA231 (*n*=3) and SKBR3 (*n*=3) cells. Graphs below image panels represent densitometric quantification of protein bands. Protein bands were normalized by calculating the POPDC1/β-actin ratio. Normalized ratios were subsequently expressed as a ratio relative to control bands (no siRNA) to determine protein fold changes. (**B**) Suppression of POPDC1 with POPDC1 siRNA significantly promoted cell migration in MCF7 (*n*=5), MDA231 cells (*n*=5) and SKBR3 cells (*n*=5). Boyden chamber assay was performed over a 3-h incubation period to allow cell migration across the polycarbonate membrane; scale bars = 250 μm. (**C**) Suppression of POPDC1 with POPDC1 siRNA significantly promoted cell proliferation in MDA231 cells (*n*=5) and SKBR3 cells (*n*=5), but not in MCF7 cells (*n*=4). Cells were transfected for 36 h prior to overnight starvation in serum-free medium and subsequent incubation in 10% AlamarBlue^®^ dye for 4 h. Comparisons of protein fold change ratios, mean % migration and mean % proliferation were conducted using ANOVA with Dunnett’s *post hoc* test. Mean values presented ± SEM; **P≤0.01, ***P≤0.001.

Suppression of POPDC1 has been shown to promote cell migration, proliferation and invasion in various human cancer cells and tumour multiplicity in mice [11,12,25]. To determine if suppression of POPDC1 affects breast cancer cell migration and proliferation, POPDC1 was suppressed with POPDC1 siRNA in MCF7, MDA231 and SKBR3 cells (Figure 3A). A significant increase in cell migration was observed following suppression of POPDC1 in MCF7, MDA231 and SKBR3 cells (Figure 3B). Furthermore, suppression of POPDC1 significantly promoted cell proliferation in the more aggressive breast cancer cell lines MDA231 and SKBR3 (Figure 3C). This dataset suggests that suppression of POPDC1 promotes cell migration and proliferation in breast cancer cells and further supports the hypothesis that dysregulation of POPDC1 promotes a more malignant phenotype in breast cancer.

### Overexpression of POPDC1 suppresses cell migration and proliferation in breast cancer cell lines

**Figure 4:**
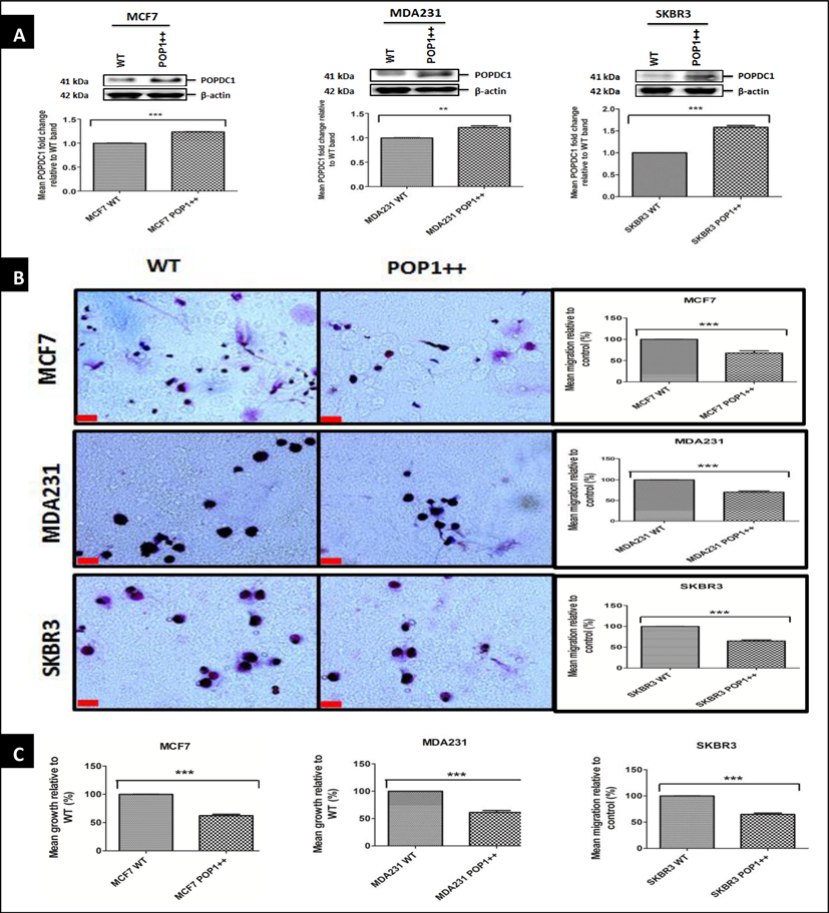
Overexpression of POPDC1 suppresses breast cancer cell migration and proliferation (**A**) Western blot analysis of POPDC1 expression in wild-type cells and cell lines stably transfected with POPDC1; MCF7 POP1++, MDA231 POP1++ and SKBR3 POP1++. Stably transfected cell lines expressed significantly higher levels of POPDC1 MCF7 (*n*=4), MDA231 (*n*=4) and SKBR3 (*n*=4). Graphs below Western blot band panels represent densitometric quantification of protein bands. Protein bands were normalized by calculating the POPDC1/β-actin ratio. Fold differences in protein expression were calculated by expressing normalized band values of POPDC1 overexpressing cell lines as a ratio to normalized band values of wild-type cell lines. (**B**) Overexpression of POPDC1 significantly suppressed cell migration in MCF7 cells (*n*=4), MDA231 (*n*=5) and SKBR3 cells (*n*=5). Boyden chamber assay was performed over a 3-h incubation period to allow cell migration across the polycarbonate membrane; scale bars = 250 μm. (**C**) Overexpression of POPDC1 significantly suppressed cell proliferation in MCF7 cells (*n*=4), MDA231 cells (*n*=5) and SKBR3 cells (*n*=5). Cells were starved in serum-free medium overnight prior to incubation in 10% AlamarBlue^®^ dye for 4 h. Comparisons of protein fold change ratios, mean % migration and mean % proliferation were conducted using an unpaired *t*-test. Mean values presented ± SEM; **P≤0.01, ***P≤0.001.

We next assessed if gain of POPDC1 function would inhibit cell migration and proliferation. Due to a lack of available POPDC1 activators or modulators, gain of function studies were conducted by overexpression of POPDC1 into MCF7, MDA231 and SKBR3 cells (Figure 4A).

Given that overexpression of POPDC1 has been shown to attenuate colorectal cancer tumour growth and metastasis in mice [12], we next asked if POPDC1 overexpression affects breast cancer cell migration and proliferation. Overexpression of POPDC1 significantly inhibited cell migration and proliferation in MCF7, MDA231 and SKBR3 cells (Figure 4B and C), consistent with our theory that POPDC1 suppresses malignant cell behaviour.

### cAMP interacts with and up-regulates POPDC1 in breast cancer cells

The Popeye domain of POPDC1 has been shown to bind cAMP with high affinity using radioligand binding assays and affinity precipitation [16]. The interaction between POPDC1 and cAMP has not been reported in breast cancer cells. To confirm if cAMP interacts with POPDC1 in breast cancer cells, we performed a pull-down assay with cAMP agarose beads. To confirm specificity of the assay, lysates were further probed for β-actin, which is known to not interact with cAMP.

**Figure 5:**
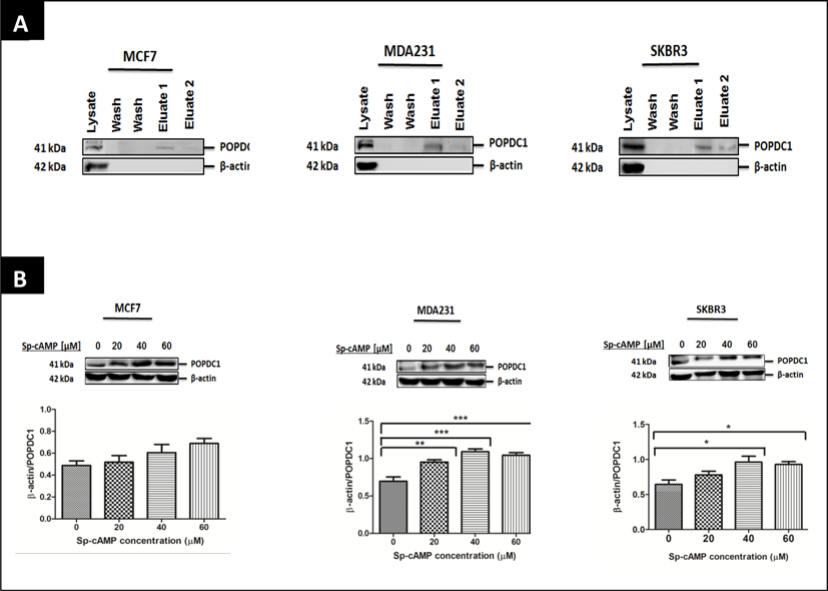
cAMP interacts with POPDC1 and up-regulates its expression in breast cancer cells (**A**) Western blot analyses of cAMP agarose pull-down assays in MCF7, MDA231 and SKBR3 cells. POPDC1 was detected at high levels in the first eluted fraction (eluate 1) and at very low levels in the second eluted fraction (eluate 2) in MCF7 (*n*=3), MDA231 (*n*=3) and SKBR3 (*n*=3) cells confirming protein interaction between POPDC1 and cAMP. β-Actin, the negative control, was detected in the unbound protein fraction but not in the eluted fractions, confirming specificity of the assay. (**B**) Western blot analysis of the effects of 20, 40 and 60 µM Sp-8-Br-cAMPS on the expression of POPDC1 and CREB following 1 h treatment duration in MCF7 (*n*=3), MDA231 (*n*=3) and SKBR3 (*n*=3) cells. Graphs below Western blot band panels represent densitometric quantification of POPDC1 bands as a ratio to corresponding β-actin bands. Comparisons of normalized β-actin/POPDC1 ratios were conducted using ANOVA with Dunnett’s *post hoc* test. Mean values presented ± SEM; *P≤0.05, **P≤0.01, ***P≤0.001.

POPDC1 was detected in the first and second eluted fractions of cAMP agarose pull-down assays in MCF7, MDA231 and SKBR3 cells. The eluted fractions contain proteins that interact with cAMP. Presence of POPDC1 in the eluted fraction confirms that POPDC1 indeed interacts with cAMP in MCF7, MDA231 and SKBR3 cells, and is pulled out of the lysate using immobilized cAMP (Figure 5A). Furthermore, the negative control β-actin was detected in the lysate fraction containing unbound proteins, but not in the eluted fractions confirming that the assay specifically eluted cAMP bound proteins. To assess the effects of cAMP on POPDC1 expression in breast cancer cells, we next asked if up-regulation of cAMP with the cAMP analogue Sp-8-Br-cAMPS can affect the levels of POPDC1. The effects of a range of Sp-8-Br-cAMPS concentrations were assessed over 1 h treatment duration (Figure 5B). Detection of POPDC1 was up-regulated by 40 and 60 µM Sp-8-Br-cAMPS in MCF7 cells, significantly up-regulated by 20, 40 and 60 µM Sp-8-Br-cAMPS in MDA231 cells and by 40 and 60 µM Sp-8-Br-cAMPS in SKBR3 cells. To confirm activation of the cAMP pathway, we assessed if 20, 40 and 60 µM Sp-8-Br-cAMPS affects the expression of phospho-CREB, another molecule that functions downstream of cAMP. Treatment with different Sp-8-Br-cAMPS concentrations increased the expression levels of phospho-CREB in MCF7, MDA231 and SKBR3 cells confirming activation of cAMP signalling (Figure 5B). This dataset confirms that cAMP indeed interacts with POPDC1 and can increase its presence and stability in breast cancer cells.

### cAMP inhibits cell migration and proliferation in breast cancer cells

**Figure 6:**
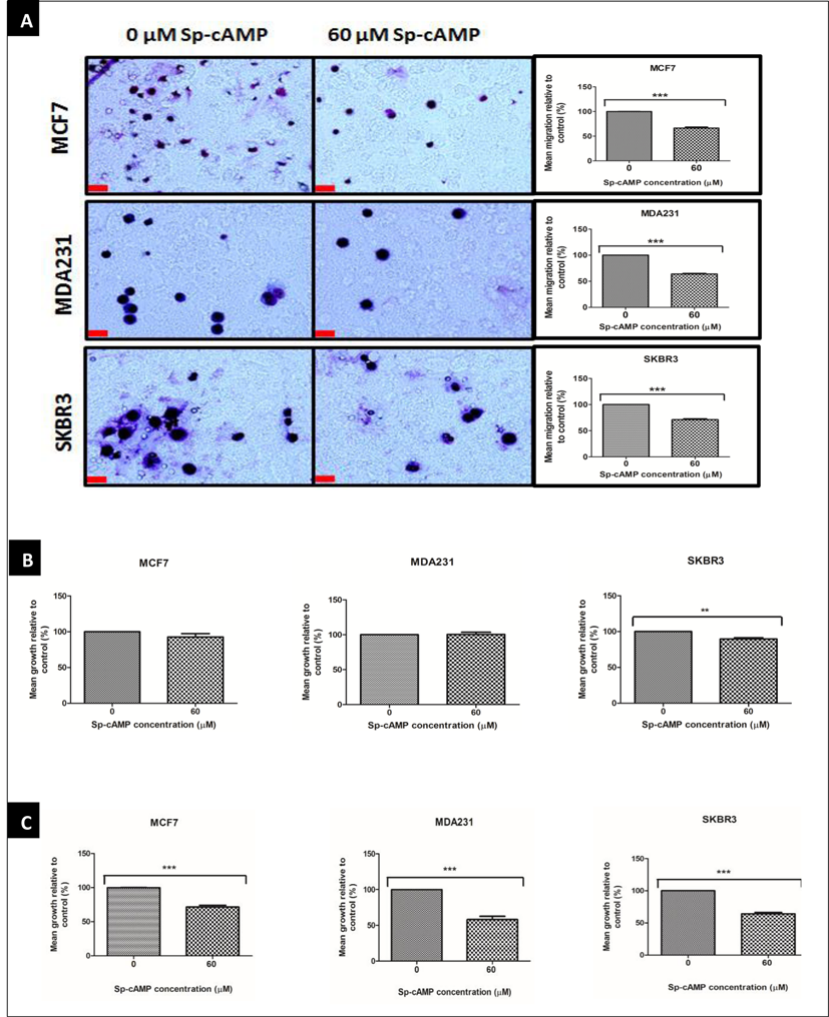
Sp-8-Br-cAMPS inhibits cell migration and proliferation in breast cancer cell lines (**A**) Boyden chamber assay analysis of the effects of 60 µM Sp-8-Br-cAMPS on breast cancer cell migration in MCF7 (*n*=4), MDA231 (*n*=4) and SKBR3 (*n*=4) cells. Boyden chamber assay was performed over a 3-h incubation period to allow cell migration across the polycarbonate membrane; scale bars = 250 μm. (**B**) Alamar Blue assay analysis of the effects of 60 µM Sp-8-Br-cAMPS on MCF7 (*n*=4), MDA231 (*n*=4) and SKBR3 (*n*=4) cell proliferation. Cells were starved in serum-free medium overnight prior to drug treatment. (**C**) Alamar Blue assay analysis of the effects of a two-phase treatment with 60 µM Sp-8-Br-cAMPS on MCF7 (*n*=5), MDA231 (*n*=5) and SKBR3 (*n*=5) cell proliferation. Cells were starved in serum-free medium overnight prior to drug treatment. Cells were subsequently treated with 60 µM Sp-8-Br-cAMPS for 1 h prior to treatment replacement with a fresh 60 µM Sp-8-Br-cAMPS solution and a further 24-h incubation. Alamar Blue assays were performed by incubating cells in 10% AlamarBlue^®^ dye (diluted in serum-free medium) for 4 h. Mean % migration and mean % proliferation values were compared using an unpaired *t*-test. Mean values presented ± SEM; **P≤0.01, ***P≤0.001.

Increasing intracellular levels of cAMP has been shown to inhibit breast cancer cell migration and invasion [22,23]. We asked if increasing intracellular levels of cAMP with 60 µM Sp-8-Br-cAMPS affects cell migration and proliferation in MCF7, MDA231 and SKBR3 cells. Sp-8-Br-cAMPS (60 µM) significantly inhibited cell migration in MCF7, MDA231 and SKBR3 cells (Figure 6A) and cell proliferation in SKBR3 cells (Figure 6B). However, no effect was observed on MCF7 and MDA231 cell proliferation. We hypothesized that the initial treatment with Sp-8-Br-cAMPS served to up-regulate the expression of POPDC1, but that insufficient concentrations then remained to actually act on the up-regulated protein due to the gradual degradation of the analogue. Therefore, we applied a further 60 µM after an hour to see what functional effects were evident when POPDC1 expression was raised. Following this two-phase treatment regimen, significant inhibition of cell proliferation was observed in MCF7, MDA231 and SKBR3 cells (Figure 6C). Taken together this dataset suggests that increasing intracellular levels of cAMP inhibits cell migration and proliferation, and that high cellular POPDC1 expression interacts with cAMP to inhibit cell proliferation. It also suggests that cAMP control of cell migration and proliferation varies in sensitivity suggesting mechanistically diverse effects.

### Suppression of POPDC1 is overcome by SP-8-Br-cAMPS and this rescues inhibited cell migration and proliferation

**Figure 7:**
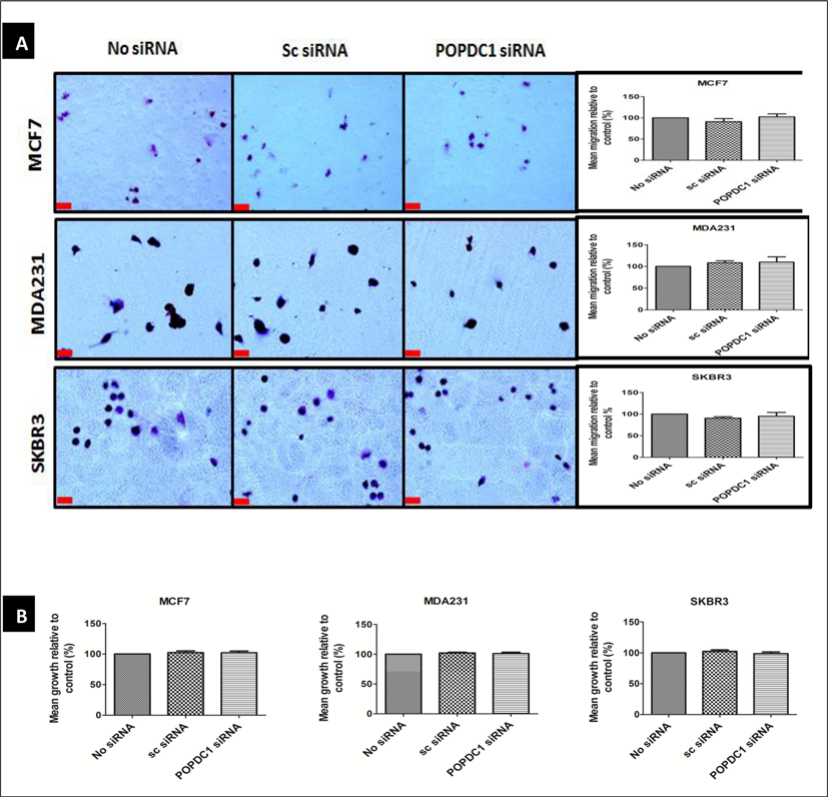
Suppression of POPDC1 is overcome by SP-8-Br-cAMPS and rescues inhibited cell migration and proliferation in breast cancer cells (**A**) Boyden chamber assay analysis of the effects of 60 µM Sp-8-Br-cAMPS on MCF7 (*n*=4), MDA231 (*n*=4) and SKBR3 (*n*=4) breast cancer cell migration following POPDC1 suppression with siRNA. Boyden chamber assay was performed over a 3-h incubation period to allow cell migration across the polycarbonate membrane; scale bars = 250 μm. (**B**) Alamar Blue assay analysis of the effects a two-phase 60 µM Sp-8-Br-cAMPS treatment on the proliferation of MCF7 (*n*=5), MDA231 (*n*=5) and SKBR3 (*n*=5) cells following POPDC1 suppression with siRNA. Cells were transfected for 36 h and starved overnight prior to drug treatment. Cells were treated with 60 µM Sp-8-Br-cAMPS treatment for 1 h prior to treatment replacement with a fresh 60 µM Sp-8-Br-cAMPS treatment solution and 24-h incubation. Alamar Blue assay was performed by incubating cells in 10% AlamarBlue^®^ dye for 4 h. Mean % migration and mean % proliferation values were compared using an unpaired *t*-test. Mean values presented ± SEM.

Although the dataset so far suggests that cAMP-mediated inhibition of cell migration and proliferation is potentially facilitated via POPDC1 signalling, cAMP-mediated signals could be facilitated via other cAMP-binding molecules such as POPDC2 and POPDC3. It is therefore essential to determine whether cAMP-mediated suppression of breast cancer cell migration and proliferation is indeed dependent on cAMP-induced increase in POPDC1 protein levels. Hence, we next asked if the POPDC1 suppression attenuates cAMP-mediated breast cancer cell migration and proliferation in these cell lines. To test this, the effects of 60 µM Sp-8-Br-cAMPS on breast cancer cell migration and proliferation were assessed following POPDC1 knockdown in MCF7, MDA231 and SKBR3 cells (Figure 7).

POPDC1 knockdown failed to alter cAMP-induced cell migration and proliferation in MCF7, MDA231 and SKBR3 cells (Figure 7A and B) with migration and proliferation levels unchanged relative to baseline. This confirms that cAMP-mediated inhibition of breast cancer cell migration and proliferation depends on high cellular POPDC1 protein levels and is in line with the present finding that cAMP upregulates POPDC1, and does so to the extent that it overcomes the siRNA mediated downregulation induced in Figure 6 and 7to rescue migration and proliferation to control levels.

## Discussion

In the present study, we have shown that POPDC1 is expressed in breast cells, but the expression is significantly suppressed in aggressive MDA231 and SKBR3 breast cancer cells, in comparison with non-malignant MCF10A breast cells (Figure 1). This is consistent with suppression of POPDC1 protein and mRNA observed in various cancers including gastric cancer, colorectal cancer and hepatocellular carcinoma [11–13].

The present study has further demonstrated differential POPDC1 localization in breast cancer cells in comparison with non-malignant breast cells. Cell membrane localization of POPDC1 is reduced in MDA231 and SKBR3 cells in comparison with localization of POPDC1 to the cell membrane in normal MCF10A breast cells (Figure 2). We hypothesize that suppression of POPDC1 expression in breast cancer cells prevents the POPDC1 functioning at the cell membrane. Suppression of POPDC1 on the cell membrane has been shown to impair POPDC1-mediated tight junction maintenance in epithelial cells [21]. We postulate that the absence of functional POPDC1 dimers on breast cancer cell membranes potentially leads to a reduction in POPDC1-mediated tight junction maintenance thereby promoting cell migration.

We have further demonstrated the functional relevance of POPDC1 dysregulation in breast cancer. Suppression of POPDC1 significantly promotes a malignant phenotype in breast cancer by promoting cell migration in MCF7, MDA231 and SKBR3 cells, and cell proliferation in the more aggressive MDA231 and SKBR3 cells (Figure 3B and C). Additionally, the overexpression of POPDC1 reverts cells to a less malignant phenotype by significantly inhibiting cell migration and proliferation in MCF7, MDA231 and SKBR3 breast cancer cells. Taken together with data from other studies which show that the suppression of POPDC1 promotes cell migration and invasion in glioblastoma, hepatocellular carcinoma, gastric cancer and colorectal cancer [12,13,25,27], these data provide strong evidence to support the hypothesis that the dysregulation of POPDC1 promotes malignant phenotypes in breast cancer. Loss of POPDC1 expression has been shown to not only promote malignant behaviour in cancer, but also promote cardiac and skeletal muscle pathologies, which are inhibited or partially reversed by gain of POPDC1 function [14,16,19]. These suggest that POPDC1 could potentially be therapeutically targeted in a similar manner to treat various diseases. Therapeutic targeting to prevent POPDC1 dysregulation or to promote gain of POPDC1 function can potentially be achieved in various ways. Firstly, gain of function could be achieved with small molecules that bind to the Popeye domain to induce protein stabilization. Secondly, truncation of the protein has been shown to prevent POPDC1-mediated tight junction maintenance by preventing localization of POPDC1 to the cell membrane [21]. Loss of POPDC1 function can therefore potentially be prevented by targeting mutations that prevent protein localization to the cell membrane or stabilizing the protein in the membrane.

The present study has further shown that cAMP interacts with POPDC1 and up-regulates its expression in MCF7, MDA231 and SKBR3 breast cancer cells (Figure 5A and B). Furthermore cAMP significantly inhibited cell migration in MCF7, MDA231 and SKBR3 cells (Figure 6A), consistent with data from other studies showing that cAMP inhibits cell migration and invasion in breast cancer cells [22,23]. However, significant inhibition of cell proliferation in MCF7 and MDA231 cells was only observed following a two-phase treatment regimen with 60 µM Sp-8-Br-cAMPS (Figure 6C). The initial Sp-8-Br-cAMPS treatment served to up-regulate the expression of POPDC1, with the subsequent treatment permitting the functional consequences of Sp-8-Br-cAMPS binding to elevated POPDC1 expression to be measured. This suggests cAMP-mediated cell proliferation is facilitated via a mechanism that at least in part involves the up-regulation of POPDC1. The hypothesis that cAMP-mediates inhibition of breast cancer cell migration and proliferation via POPDC1 signalling is further strengthened by restoration of the cell migration and proliferation in MCF7, MDA231 and SKBR3 cells by elevating cAMP following POPDC1 knockdown (Figure 7A and B) which suggests that these effects are dependent upon a cAMP-induced increase in POPDC1 levels. Furthermore, it suggests that migration does not require the up-regulation of POPDC1 as it was inhibited by a single treatment. This is consistent with a mechanism that involves direct action of Sp-8-Br-cAMPS in stabilizing the existing POPDC1 protein being sufficient to reduce migration. It further suggests a more complex and less direct mechanism involved in cAMP potentially exerting control of proliferation via POPDC1. This firstly requires up-regulation of the protein, and subsequent interaction with cAMP to influence proliferation perhaps via a downstream moiety. This molecular diversity is a novel and interesting finding that may further extend the therapeutic potential of POPDC1 as a target.

In conclusion, POPDC1 represents a druggable target that can potentially be manipulated to inhibit breast cancer cell migration and proliferation. In particular, this seems possible by binding of the cAMP-binding Popeye domain, which seems to establish protein function in the plasma membrane. As a unique molecular domain, molecules that can interact in a cAMP-mimicking manner seem like exciting candidates for investigation going forward. However, mechanisms by which POPDC1 regulates cell migration and proliferation in breast cancer need to be clearly elucidated in order to permit development of directed therapeutics to take advantage of POPDC1 and its diverse molecular role.

## Highlights

- Expression and cell membrane localization of POPDC1 is reduced in breast cancer cells.
- Suppression of POPDC1 promotes breast cancer cell migration and proliferation.
- Overexpression of POPDC1 inhibits breast cancer cell migration and proliferation.
- cAMP interacts with, and up-regulates POPDC1 in breast cancer..

## Abbreviations

BVES: blood vessel epicardial substance
DMEM: Dulbecco’s Modified Eagle’s medium
ER: oestrogen receptor
HER2: human epidermal growth factor 2 receptor
PLL: poly-l-lysine
POPDC1: Popeye domain-containing protein 1
PR: progesterone receptor
RIPA: Radioimmunoprecipitation Assay

## References

[1] Globocan W. Estimated cancer incidence, mortality and prevalence worldwide in 2012. 2014-01-09)[2014-07-01], http://globocan.iarc.fr/Pages/fact_sheets_population.Aspx

[2] Ferrer-Soler L., Vazquez-Martin A., Brunet J., Menendez J.A., De Llorens R. and Colomer R. (2007) An update of the mechanisms of resistance to EGFR-tyrosine kinase inhibitors in breast cancer: Gefitinib (iressa™)-induced changes in the expression and nucleo-cytoplasmic trafficking of HER-ligands (review). Int. J. Mol. Med. 20, 3 17549382

[3] Normanno N., Luca A.D., Maiello M.R., Campiglio M., Napolitano M., Mancino M. (2006) The MEK/MAPK pathway is involved in the resistance of breast cancer cells to the EGFR tyrosine kinase inhibitor gefitinib. J. Cell. Physiol. 207, 420–427 16419029 10.1002/jcp.20588

[4] Mirzoeva O.K., Das D., Heiser L.M., Bhattacharya S., Siwak D., Gendelman R. (2009) Basal subtype and MAPK/ERK kinase (MEK)-phosphoinositide 3-kinase feedback signaling determine susceptibility of breast cancer cells to MEK inhibition. Cancer Res. 69, 565–572 19147570 10.1158/0008-5472.CAN-08-3389 PMC2737189

[5] Li C., Iida M., Dunn E.F., Ghia A.J. and Wheeler D.L. (2009) Nuclear EGFR contributes to acquired resistance to cetuximab. Oncogene 28, 3801–3813 19684613 10.1038/onc.2009.234 PMC2900381

[6] Brenton J.D., Carey L.A., Ahmed A.A. and Caldas C. (2005) Molecular classification and molecular forecasting of breast cancer: ready for clinical application? J. Clin. Oncol. 23, 7350–7360 16145060 10.1200/JCO.2005.03.3845

[7] O’Donovan N. and Crown J. (2007) EGFR and HER-2 antagonists in breast cancer. Anticancer Res. 27, 1285–1294 17593621

[8] Holliday D.L. and Speirs V. (2011) Choosing the right cell line for breast cancer research. Breast Cancer Res. 13, 1 10.1186/bcr2889 PMC3236329 21884641

[9] Han P., Fu Y., Luo M., He J., Liu J., Liao J. (2014) BVES inhibition triggers epithelial-mesenchymal transition in human hepatocellular carcinoma. Dig. Dis. Sci. 59, 992–1000 24442236 10.1007/s10620-013-2992-3

[10] Amunjela J.N. and Tucker S.J. (2016) POPDC proteins as potential novel therapeutic targets in cancer. Drug Discov. Today 21, 1920–1927 27458118 10.1016/j.drudis.2016.07.011

[11] Han P., Fu Y., Liu J., Wang Y., He J., Gong J. (2015) Netrin-1 promotes cell migration and invasion by down-regulation of BVES expression in human hepatocellular carcinoma. Am. J. Cancer Res. 5, 1396 26101705 PMC4473318

[12] Parang B., Kaz A.M., Barrett C.W., Short S.P., Ning W., Keating C.E. (2017) BVES regulates c-Myc stability via PP2A and suppresses colitis-induced tumourigenesis. Gut 66, 852 28389570 10.1136/gutjnl-2015-310255 PMC5385850

[13] Kim M., Jang H.R., Haam K., Kang T.W., Kim J.H., Kim S.Y. (2010) Frequent silencing of popeye domain-containing genes, BVES and POPDC3, is associated with promoter hypermethylation in gastric cancer. Carcinogenesis 31, 1685–1693 20627872 10.1093/carcin/bgq144

[14] Schindler R.F. and Brand T. (2016) The Popeye domain containing protein family–A novel class of cAMP effectors with important functions in multiple tissues. Prog. Biophys. Mol. Biol. 120, 28–36 26772438 10.1016/j.pbiomolbio.2016.01.001 PMC4821176

[15] Amunjela J.N. and Tucker S.J. (2016) POPDC proteins as potential novel therapeutic targets in cancer. Drug Discov. Today, 21, 1920–1927, 21 27458118 10.1016/j.drudis.2016.07.011

[16] Froese A., Breher S.S., Waldeyer C., Schindler R.F., Nikolaev V.O., Rinné S. (2012) Popeye domain containing proteins are essential for stress-mediated modulation of cardiac pacemaking in mice. J. Clin. Invest. 122, 1119 22354168 10.1172/JCI59410 PMC3287222

[17] Schindler R.F., Poon K.L., Simrick S. and Brand T. (2012) The popeye domain containing genes: essential elements in heart rate control. Cardiovasc. Diagn. Ther. 2, 308 24282731 10.3978/j.issn.2223-3652.2012.12.01 PMC3839166

[18] Andrée B., Hillemann T., Kessler-Icekson G., Schmitt-John T., Jockusch H., Arnold H. (2000) Isolation and characterization of the novel popeye gene family expressed in skeletal muscle and heart. Dev. Biol. 223, 371–382 10882522 10.1006/dbio.2000.9751

[19] Simrick S., Schindler R.F., Poon K. and Brand T. (2013) Popeye domain-containing proteins and stress-mediated modulation of cardiac pacemaking. Trends Cardiovasc. Med. 23, 257–263 23562093 10.1016/j.tcm.2013.02.002 PMC4916994

[20] Andrée B., Fleige A., Hillemann T., Arnold H., Kessler-Icekson G. and Brand T. (2002) Molecular and functional analysis of popeye genes: a novel family of transmembrane proteins preferentially expressed in heart and skeletal muscle. Exp. Clin. Cardiol. 7, 99–103 19649231 PMC2719169

[21] Russ P.K., Pino C.J., Williams C.S., Bader D.M., Haselton F.R. and Chang M.S. (2011) Bves modulates tight junction associated signaling. PLoS One 6, e14563 21283798 10.1371/journal.pone.0014563 PMC3024319

[22] Spina A., Di Maiolo F., Esposito A., Sapio L., Chiosi E., Sorvillo L. (2012) cAMP elevation down-regulates β 3 integrin and focal adhesion kinase and inhibits leptin-induced migration of MDA-MB-231 breast cancer cells. BioResearch Open Access 1, 324–332 23515360 10.1089/biores.2012.0270 PMC3559230

[23] Bianco C., Tortora G., Baldassarre G., Caputo R., Fontanini G., Chine S. (1997) 8-chloro-cyclic AMP inhibits autocrine and angiogenic growth factor production in human colorectal and breast cancer. Clin. Cancer Res. 3, 439–448 9815703

[24] Ramage A., Langdon S., Ritchie A., Burns D. and Miller W. (1995) Growth inhibition by 8-chloro cyclic AMP of human HT29 colorectal and ZR-75-1 breast carcinoma xenografts is associated with selective modulation of protein kinase A isoenzymes. Eur. J. Cancer 31, 969–973 10.1016/0959-8049(95)00190-5 7646930

[25] Amunjela J. and Tucker S. (2016) Loss of popdc protein expression promotes a more malignant phenotype in glioblastoma and breast cancer cells. Eur. J. Cancer 61, S46

[26] Osler M.E., Chang M.S. and Bader D.M. (2005) Bves modulates epithelial integrity through an interaction at the tight junction. J. Cell Sci. 118, 4667–4678 16188940 10.1242/jcs.02588

[27] Williams C.S., Zhang B., Smith J.J., Jayagopal A., Barrett C.W., Pino C. (2011) BVES regulates EMT in human corneal and colon cancer cells and is silenced via promoter methylation in human colorectal carcinoma. J. Clin. Invest. 121, 4056–4069 21911938 10.1172/JCI44228 PMC3195453

